# Augmented and prematurely occurring H-reflex recovery curve shows enhanced spinal network excitability in ALS patients when employing the double stimulation paradigm

**DOI:** 10.3389/fneur.2026.1790581

**Published:** 2026-04-09

**Authors:** Sebastian A. Wölk, Johannes Prudlo, Uwe Walter, Robert Patejdl

**Affiliations:** 1 Oscar Langendorff Institute of Physiology, Rostock University Medical Center, Rostock, Germany; 2 Department of Neurology, Rostock University Medical Center, Rostock, Germany; 3 Department of Medicine, Health and Medical University Erfurt, Erfurt, Germany

**Keywords:** amyotrophic lateral sclerosis, corticospinal, electrodiagnosis, H-reflex, H-reflex recovery curve, recurrent inhibition, Renshaw cell, spinal network excitability

## Abstract

Amyotrophic Lateral Sclerosis (ALS) is a fatal neurodegenerative disorder in which motoneuron loss in the brain and spinal cord induces complex neuroplastic changes. Although these alterations hold considerable potential for clinical diagnosis and disease monitoring, they remain underutilized due to the lack of sensitive and non-invasive assessment methods. The H-reflex is a monosynaptic spinal reflex arc and represents the neurophysiological analog of the Achilles tendon reflex. A modified H-reflex double-stimulation paradigm enables differentiation of the temporal dynamics of spinal inhibitory and excitatory mechanisms. In ALS, this approach may provide clinically relevant insights into motor system dysfunction. Furthermore, this approach may contribute to a better understanding of the pathophysiology of spinal network plasticity associated with this disease, reflecting the complex interplay between spinal and supraspinal pathways. We assessed H-reflex recovery in 15 ALS patients and 82 non-ALS subjects, including 12 age-matched healthy controls (HC). The protocol included 14 interstimulus intervals (ISI) within a timeframe of up to one second. In contrast to the HCs, ALS patients exhibited recovery at ISIs of 30 or 50 ms. At interstimulus intervals ranging from 50 to 200 ms, the extent of recovery was significantly elevated in the ALS group compared to the age-matched HCs. ALS patients thus demonstrate heightened spinal network excitability as evidenced by an augmented and prematurely occurring H-reflex recovery measurement. These alterations likely reflect changes in neuronal network activity and can be attributed to modifications in both segmental spinal circuits and supraspinal regulatory pathways.

## 1 Introduction

Amyotrophic lateral sclerosis (ALS) is a progressive neurodegenerative disorder characterized by degeneration of both upper and lower motor neurons in the cortex, brainstem, and spinal cord (1). ALS is diagnosed clinically and requires evidence of both upper and lower motor neuron involvement in at least one region in the central nervous system (bulbar, cervical, thoracic, or lumbosacral) (2). Muscle atrophy or fasciculations are signs of lower motor neuron damage (3), whereas spasticity and pathologically exaggerated reflexes indicate upper motor neuron impairment (4).

Identifying upper and lower motor neuron signs in at least one region is a hallmark of “classical” ALS (in contrast to some of its variant forms). However, accurately evaluating upper motor neuron signs in patients with concomitant muscle atrophy can be challenging, potentially delaying diagnostic confirmations (5). Therefore, additional objective diagnostic methods, beyond examiner-dependent clinical evaluations, are necessary to facilitate earlier and more accurate diagnosis (6, 7).

The H-reflex assessment, a monosynaptic reflex loop in the spinal cord, offers a measurable correlate of spinal reflex changes that can be modulated by upper motor neuron (5). In contrast to the mechanically-induced T-reflex triggered by a strike to the Achilles tendon, the H-reflex is elicited through transcutaneous nerve stimulation of a mixed nerve. Typically, the tibial nerve is stimulated at the knee bend and recorded at the soleus muscle. Rather than representing a simple index of motoneuron pool excitability, the H-reflex is more appropriately conceptualized as a measure of the functional efficacy with which activated Ia afferents evoke motoneuron discharge (8–10). This transmission efficacy reflects a dynamic interplay between presynaptic mechanisms regulating Ia afferent input and postsynaptic processes governing motoneuron responsiveness. Therefore, the physiological interpretation of the H-reflex requires careful consideration of both methodological and neurophysiological factors. Moreover, pronounced intra- and inter-individual variability in reflex responses necessitates standardized stimulation protocols and appropriate normalization procedures to ensure valid comparisons across experimental conditions and recording sessions (11, 12).

A variation of the classical H-reflex assessment is the evaluation of the H-reflex recovery curve. This approach uses a double-stimulation protocol to examine spinal excitability by analyzing the reciprocal interaction between two identical stimuli delivered at varying interstimulus intervals (ISI) (13).

In healthy individuals, the H-reflex recovery curve follows a characteristic and reproducible pattern. Following initial reflex suppression at short ISIs (12–80 ms), motoneuron recovery typically occurs between ISIs of 80 and 300 ms (14), followed by a phase characterized by a slight decrease in recovery values at ISIs from 300 to 900 ms (15). Motoneuron recovery is expected to be complete following intervals of three and eight seconds, respectively (14, 16). The shape and timing of the H-reflex recovery curve provide valuable insights into neuronal network activity and the dynamic balance between excitatory and inhibitory inputs to spinal motor neurons. This curve is shaped by multiple factors whose strength and contribution can vary, depending on the ISI. The H-reflex results from a complex interplay between spinal and supraspinal mechanisms (16). For short interval times of less than 110 ms, recurrent inhibition (RI) is regarded as a significant contributor to the strong inhibition of the test stimulus, acting as negative feedback inhibition through Renshaw cells (17). Besides RI, spinal network activity is also influenced by presynaptic inhibition, post-activation depression, and both autogenic and reciprocal inhibition mechanisms (18, 19). Supraspinal influences have been identified, particularly for ISI times exceeding 200 ms (20).

In ALS patients, changes in inhibitory circuits are observed (21). At the spinal level, alterations in recurrent inhibition are evident (22, 23), while at the central level, modifications in descending motor pathways have been identified (24). Recent evidence indicates that these changes are not merely secondary effects of motor neuron loss but reflect a systemic failure of complex spinal network organization (25, 26).

Although H-reflex recovery measurements have yet to be widely implemented in clinical practice, studies have revealed abnormalities in patients with conditions such as spasticity (27, 28), extrapyramidal disorders (29), and cerebellar damage (30). In cases of disease remission or improvement, H-reflex recovery is possible, for example in Parkinson’s disease following thalamotomy or levodopa therapy (29, 31). By contrast, in ALS, H-reflex recovery measurements seem to be rarely investigated.

This study aims to investigate changes in neuronal network activity utilizing a comprehensive ISI range in ALS patients, focusing particularly on upper motor neuron involvement by applying a double-stimulation paradigm and recording the complete H-reflex recovery curve up to an ISI of one second.

## 2 Materials and methods

### 2.1 Study population and patient selection

The ALS group in which H-recovery could be studied included 15 patients, of whom 13 met the criteria for classic ALS with combined upper and lower motor neuron involvement, with two diagnosed with primary lateral sclerosis. Most had a disease duration of under 1.5 years. Demographic and disease-relevant clinical data were collected, including symptom onset, onset region, ALSFRS-R score, limb atrophy, reflex status, and the degree of fasciculations and spasticity.

In eight additional ALS patients, no H-reflex recovery curve could be recorded. Disease duration did not differ significantly between this subgroup and the main cohort. These patients predominantly exhibited lower limb muscle atrophy and reduced tendon reflexes. Of these, six displayed diminished or absent Achilles tendon reflexes accompanied by mild to severe leg muscle atrophy. The data and characteristics for individuals in whom a measurement was successfully conducted are presented in Table 1.

**Table 1:** Overview of the demographics characteristics of the groups.

Characteristics	ALS (*n* = 15)	Age-matched controls (*n* = 12)	*p*	Mixed controls (*n* = 82^*^)	*p*
Sex (M:F)	10:5	6:6	0.452 Fi.	40:42	0.265 Fi.
Age (years)	64.6 ± 10.8	68.1 ± 7.6	0.409 t	29.7 (5.8)	<0.001 U
Body height (m)	1.74 ± 0.1	1.72 ± 0.1	0.520 t	1.75 (0.1)	0.840 t
Body Mass (kg)	73 ± 11	84 ± 25	0.157 t	72.0 (18.5)	0.382 U
BMI (kg/m^2^)	24 ± 2.6	28 ± 6.2	0.064 U	23.2 (3.9)	0.155 U
Crampi index score	102 (46.5)	2 (2.3)	0.003 U	0.7 (0)	<0.001 U
ALSFRS-R	38 ± 6				
Disease duration (months)	17.4 ± 14				
Onset region (bulb:C, Th, L/S)	4:11				
Modified Ashworth score	1 ± 1.4				
At least mild atrophies in					
Arms (Yes:No)	10:5				
Legs (Yes:No)	4:11				
At least mild fasciculations in					
Arms (Yes:No)	6:9				
Legs (Yes:No)	7:8				
Lively or heightened reflexes in					
Arms (Yes:No)	10:5				
Legs (Yes:No)	8:7				

BMI, Body-Mass-Index; bulb, bulbar; C, cervical; Th, thoracic; L/S, lumbosacral, modified Ashworth score ranging from Grade 0 (no increased tone) to Grade 4 (significantly increased tone) (61), Cramp Index score = number of cramps per month x severity (1–4) x duration (1–3) (62). The *p*-values represent the statistical comparisons between the ALS cohort and age-matched controls in addition to between the ALS cohort and mixed controls as determined by Fisher’s exact test (Fi.), the *t*-test (*t*), and the Mann–Whitney U test (U). The value in parentheses represents the interquartile range in cases where the group does not follow a normal distribution.

^*^The 12 age-matched controls were considered as part of the mixed control group.

The ALS results were compared to two other control groups. First, a mixed control group in which 82 subjects were included regardless of age, most being medical students. Second, 12 subjects without motor neuron disease were recruited as age-matched controls. While the majority of the control subjects were healthy volunteers, one-third consisted of individuals hospitalized for a transient ischemic attack. Inclusion for these participants was strictly contingent upon the absence of structural neurological lesions in cerebral imaging (magnetic resonance imaging including diffusion weighted sequences in 2 planes, T2 and T1 weighted sequences) and the lack of clinical symptoms or signs suggestive of motor neuron disease, upper motor neuron involvement, or clinically manifest polyneuropathy. The 12 age-matched controls were considered as part of the mixed control group.

The inclusion of subjects or patients proceeded after obtaining informed consent from the individual or their legal representative, all of which have been documented in writing.

### 2.2 Measurement of the H-reflex and its recovery curve

We adopted the classical H-reflex setup, for which the maximal amplitudes of the M-wave and H-wave are compared to determine the proportion of motor neurons that can be maximally activated by the reflex out of the total pool of potentially excitable motor neurons (32). In this approach, a conditioning stimulus is applied just before the test stimulus. The H-wave amplitude of the test stimulus (H2) as a percentage of the H-wave amplitude of the conditioning stimulus (H1) is referred to as recovery and depends on the temporal interval between the two stimuli and can vary between 0 and 100%, recovery = $100*(\frac{H2}{H1})$ (14, 33).

### 2.3 Experimental protocol

The measurement of the H-reflex recovery curve was performed on the soleus muscle using the classical belly-tendon electrode arrangement after stimulation of the tibial nerve in the popliteal fossa (15). For this, subjects lie prone, supporting their head on the examination table. The leg side that exhibits a livelier triceps surae reflex during clinical examination is measured (23). Fourteen different interstimulus intervals (ISI) were investigated, specifically the ISI times of 10 ms, 30 ms, 50 ms, 70 ms, 100 ms, 150 ms, 200 ms, 250 ms, 300 ms, 350 ms, 400 ms, 500 ms, 700 ms, and 1,000 ms, aiming to depict the entire recovery process as reported previously in cohorts of healthy volunteers and patients suffering from spinal cord lesions (32, 34). The ISI times refer to the gap between the conditioning and test stimuli. There is a ten-second pause between each ISI to prevent the influence of the previous stimulation on the next ISI (35).

Stimulation of the posterior tibial nerve was performed according to the following standardization and recruitment protocol. After identifying the optimal stimulation site, stimulation intensity was systematically varied to map recruitment characteristics. Repeated stimulations were performed to establish a stable baseline and account for fluctuations in transcutaneous impedance. Stimulation intensity was adjusted to maintain the test H-reflex on the ascending limb of the recruitment curve to ensure maximum sensitivity to modulatory influences (9). We targeted an H/M-wave ratio of approximately 1.0, using identical intensities for both conditioning and test stimuli. The concomitant M-wave served as a reliable real-time indicator of the effective stimulus current (36). Continuous comparison of the M-wave responses evoked by the conditioning and test stimuli served as an internal control. To ensure data consistency, stimulation intensity was dynamically adjusted during the measurement if the M-wave amplitudes deviated from the target level.

### 2.4 Signal processing and statistical analysis

Stimulation was applied using a stimulator from Digitimer Ltd. (MultiPulse Transcranial Cortical Stimulator, Article Number: D185, Manufacturer: Digitimer Ltd., Hertfordshire, United Kingdom) with a square pulse waveform and a stimulation duration of 1 ms. Recordings were captured via a PowerLab (PowerLab 4/26, Article Number: PL2604, Manufacturer: ADInstruments, Oxford, United Kingdom) system and documented in LabChart (LabChart Version 7, Manufacturer: ADInstruments, Oxford, United Kingdom). Measurements were conducted with a 10 kHz sampling rate, a 20 mV range, band-pass filters set at 10 Hz (high-pass) and 2 kHz (low-pass), and an active mains filter. The stimulation protocol was pre-programmed and identical across all recordings.

Onset latency was defined as the first consistent deflection from the baseline preceding the primary response peak. To avoid filter-induced artifacts, all onsets were manually determined via visual inspection. Visual inspection was used as it is considered the gold standard for its high sensitivity in identifying onset events in electrophysiological signals (37). To ensure high internal consistency, all analyses were performed by a single investigator.

Statistical analyses were performed using SigmaStat (Version 3.5; Systat Software GmbH, Erkrath, Germany), IBM SPSS Statistics (Version 29; IBM Corp., Armonk, NY, USA), and Microsoft Excel (Microsoft, Redmond, WA, USA). Normal distribution of the data was assessed using the Kolmogorov–Smirnov test. For comparisons between two groups, Student’s *t*-test was used for normally distributed data (reported as mean ± SD). In cases of non-normal distributed data or unequal variances, the Mann–Whitney Rank Sum test was applied (reported as median and interquartile range [IQR]). To assess the magnitude of the observed differences, effect sizes were calculated as Cohen’s d for independent *t*-tests and *r* for Mann–Whitney U-tests. According to the widely used criteria by Cohen (38), effect sizes for d were interpreted as small (≥ 0.2), medium (≥ 0.5), and large (≥ 0.8). For the *r* statistic, values were categorized as small (≥ 0.1), medium (≥ 0.3), and large (≥ 0.5).

An analysis of covariance (ANCOVA) was applied to the 150 ms ISI data to assess group differences while adjusting for age. To analyze differences between groups across multiple ISIs, a two-way repeated measures analysis of variance (ANOVA) was performed. Partial eta-squared (ηp^2^) was calculated as a measure of effect size to estimate the proportion of variance explained by each factor. Following the benchmarks proposed by Cohen (38), ηp^2^ values were interpreted as small (≥ 0.01), medium (≥ 0.06), and large (≥ 0.14).

Correlations between H-reflex recovery measurement results and clinical data were evaluated using Spearman’s rank correlation coefficient [0.20–0.39: low; 0.40–0.59: moderate; 0.60–0.79: moderately high; and ≥ 0.80: high correlation (39)]. Additionally, raw values were transformed into Z-scores to allow for standardized comparison across different parameters. Unless specified otherwise, values from the ALS cohort were compared to those of the age-matched or mixed control groups. Statistical significance was defined as *p* < 0.05. The discriminatory power of the measured parameters was assessed using Receiver Operating Characteristic (ROC) analysis, with cut-off values determined by the Youden Index (40).

## 3 Results

### 3.1 H-reflex

Baseline H-reflex values differed between groups. In ALS patients, significant differences in M-wave latency, H-wave latency, and H-wave amplitude were observed compared to mixed controls, while only M-wave latency differed significantly from age-matched controls. The mean M-wave latency in ALS patients (5.85 ± 1.24 ms) was significantly longer than in age-matched controls (4.74 ± 0.58 ms). H-wave latency in ALS patients (34.39 ± 4.12 ms) was slightly prolonged compared to age-matched controls (33.23 ± 3.85 ms), but this difference was not statistically significant. The shortest H-wave latencies were observed in mixed controls. Variations in body height between groups were minimal and did not warrant statistical adjustment. H-wave amplitudes did not differ significantly between ALS patients (3.57 ± 2.55 mV) and age-matched controls (2.12 ± 1.10 mV) but were significantly higher in mixed controls (6.66 ± 3.11 mV). Stimulation intensities showed no significant group differences. The ALS group had the lowest average intensity at 86.60 mA (IQR 35.00 mA).

Table 2 presents the baseline values along with the associated statistical analysis for the H-reflex.

**Table 2:** Overview of the baseline values of the H-reflex.

Characteristics	ALS patients	Control 1	Control 2
	Mean (SD/*IQR*) [95% CI]		
H-reflex latency in ms	34.39 (4.12) [32.11 to 36.67]	33.23 (3.85) [30.78 to 35.67]	30.09 (*2.60*) [29.53 to 30.66]
M-wave latency in ms	5.85 (1.24) [5.16 to 6.53]	4.74 (0.58) [4.37 to 5.11]	4.81 (*0.98*) [4.62 to 5.01]
H-wave amplitude in mV	3.57 (2.55) [2.16 to 4.99]	2.12 (1.10) [1.42 to 2.82]	6.66 (3.11) [5.98 to 7.35]
M-wave amplitude in mV	2.18 (1.06) [1.59 to 2.77]	2.75 (*1.14*) [1.86 to 3.65]	5.28 (*4.85*) [4.55 to 6.01]
H-wave/ M-wave ratio	1.14 (0.75) [0.72 to 1.56]	0.80 (*0.93*) [0.44 to 1.15]	1.42 (*0.82*) [1.12 to 1.71]
Stimulation strength in mA	86.60 (*35.00*) [57.14 to 116.06]	118.17 (*38.00*) [65.05 to 171.28]	91.04 (*52.75*) [80.07 to 102.01]
The value in italics represents the interquartile range, which is provided in the absence of normally distributed data.			

Characteristics	Statistical analyses					
	ALS and Co. 1			ALS and Co. 1		
	Statistical test	*p*-value	Effect size	Statistical test	*p*-value	Effect size
H-reflex latency in ms	*t*-test	*p* = 0.458	d = 0.29	U-test	*p* **= <0.001**	*r* = 0.39
M-wave latency in ms	*t*-test	***p* = 0.009**	d = 1.10	U-test	***p* = 0.001**	*r* = 0.33
H-wave amplitude in mV	U-test	*p* = 0.164	*r* = 0.12	U-test	*p* **= <0.001**	d = 0.36
M-wave amplitude in mV	*t*-test	*p* = 0.240	d = 0.47	U-test	*p* **= <0.001**	*r* = 0.39
H-wave/ M-wave ratio	*t*-test	*p* = 0.200	d = 0.51	U-test	*p* = 0.795	*r* = 0.03
Stimulation strength in mA	U-test	*p* = 0.970	*r* = 0.32	U-test	*p* = 0.413	*r* = 0.08

The *p*-values correspond to statistical comparisons between the ALS cohort and age-matched controls (Co 1), and between the ALS cohort and mixed controls (Co 2), as assessed using the *t*-test (*t*) and the Mann–Whitney U test (U). Effect sizes were quantified as Cohen’s d for *t*-tests and *r* for Mann–Whitney U-tests.

Bold values indicate statistical significance (*p* < 0.05).

### 3.2 H-reflex recovery curve

Recovery values with increasing ISIs showed similar patterns across all groups and could be categorized into three stages: in the first stage, no recovery occured, and the test stimulus failed to elicit an H-wave; in the second dynamic stage, beginning at an ISI of 50 ms, rapid and pronounced recovery featured, with the maximum amplitude typically reached; in the third stage, starting at an ISI of 300 ms, a stable recovery level with only minor modulation up to an ISI of 1,000 ms could be observed. Recovery did not return to the peak levels seen during the dynamic stage, and complete recovery was not achieved even at an ISI of one second.

In particular, H-reflex recovery in age-matched controls was initially detected at interstimulus intervals greater than 70 ms, with most participants showing partial recovery by 100 ms. Recovery subsequently rose and reached a mean peak of 72% at an ISI of 300 ms. At longer ISIs, recovery decreased slightly, reaching 59% at an ISI of 1,000 ms. In mixed controls, the recovery dynamics were comparable, however, individual values were frequently 5 to 10% higher than those observed in age-matched controls, and recovery began earlier, at an ISI between 30 ms and 50 ms.

In ALS patients, recovery began earlier, and the maximum value also appeared to be reached sooner. At an ISI of 30 ms, an average recovery of 4% was already achieved. For ISI intervals ranging from 50 ms (mean recovery of 12%) to 200 ms (mean recovery of 83%), the elevated recovery values observed in ALS patients compared to age-matched controls were statistically significant. The average peak of recovery was reached at an ISI of 250 ms, with a value of 87%. Throughout the entire third phase, mean values were generally elevated in ALS patients; however, they were significantly greater only at an ISI of 500 ms when compared to age-matched controls. After an ISI of 1,000 ms, recovery reached 70%.

The two patients with primary lateral sclerosis did not exhibit marked differences in recovery compared to the remainder of the ALS cohort. For most parameters, z-scores deviated by less than ±1 standard deviation from the cohort mean, indicating substantial overlap in the measured values.

Potential age-related influences on recovery dynamics were investigated using an analysis of covariance for the 150 ms ISI. This specific interval was selected as the data followed a normal distribution in both control groups and demonstrated a strong effect size in the pairwise analysis for the matched controls. In the age-matched sub-cohort, the ANCOVA confirmed a significant group difference between ALS patients and controls [*F*(1, 24) = 4.848, *p* = 0.038], while age itself did not significantly impact the recovery dynamics (*p* = 0.392).

In the larger, more heterogeneous control sample, age was identified as a significant factor [*F*(1, 94) = 13.026, *p* < 0.001]. However, even after statistically controlling for this age effect, the pathological difference between the ALS group and the mixed control group remained significant [*F*(1, 94) = 5.890, *p* = 0.017]. Notably, a direct comparison without age-adjustment failed to reach significance.

To enhance control over the statistical error rate and to investigate the interaction between ISI and group classification, a two-way repeated measures analysis of variance was conducted on the data. The analysis indicated that group affiliation had a significant effect on recovery when comparing age-matched controls to the ALS cohort. In contrast, no significant group effect was found in the comparison with mixed controls. In both comparisons, the timing between stimuli showed a significant effect on recovery. However, no significant interaction between group affiliation and stimulus timing was observed in either case.

**Figure 1:**
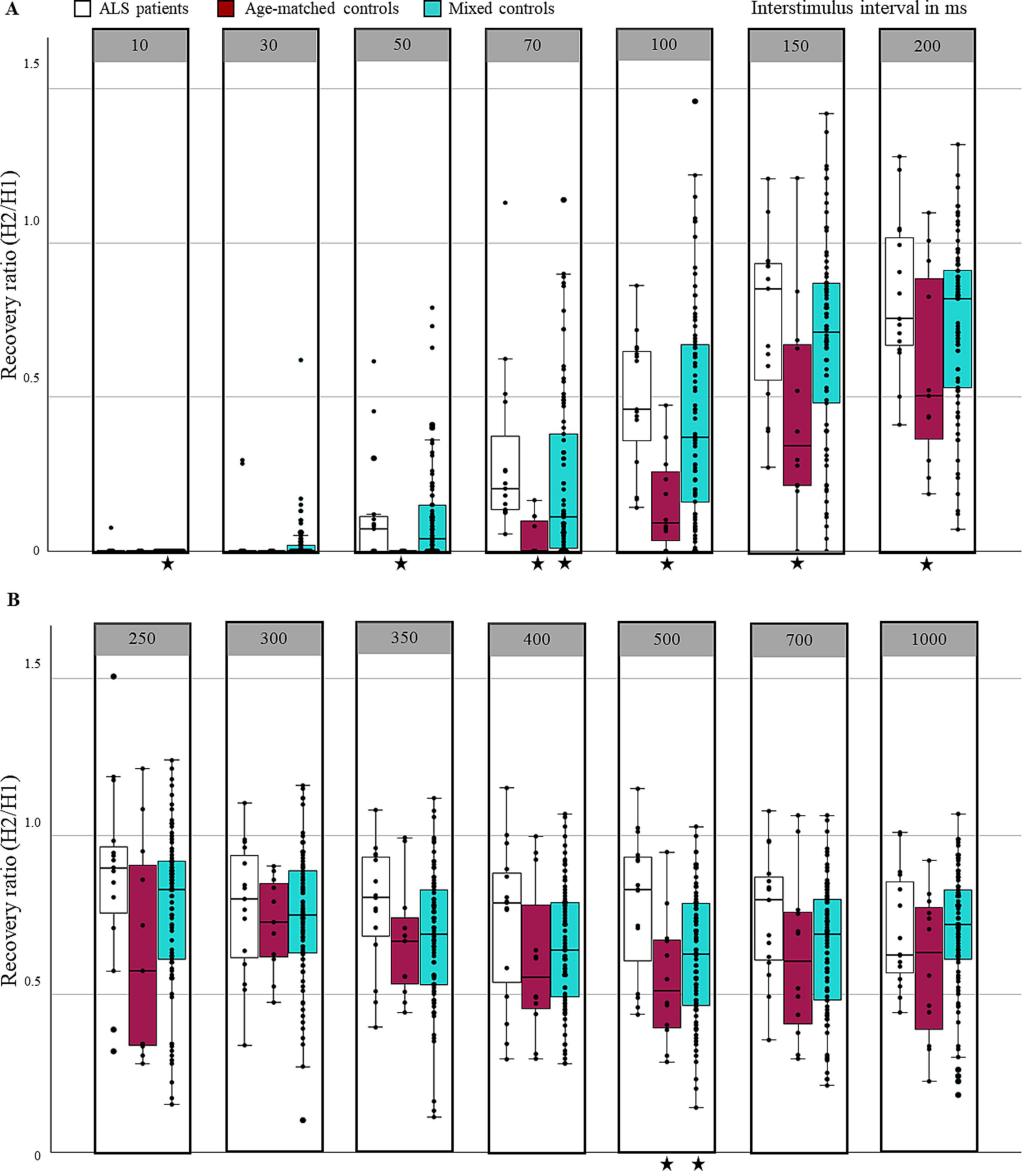
Comparison of H-reflex recovery curves: Box plots and jittered individual data across various ISIs. **(A)** H-reflex recovery for ISI up to 200 ms. **(B)** H-reflex recovery for ISI up to 1000 ms. Stars (*) denote significant differences between the ALS and control cohorts at specific ISIs (for detailed *p*-values and statistics, see Table 3).

The measurement results from all successfully conducted measurements are presented in Figure 1 while the statistical analysis comparing the results between the ALS patients and the controls is provided in Tables 3, 4.

**Table 3:** Overview of H-reflex recovery measurements across different interstimulus intervals.

ISI	ALS patients	Control 1	Control 2
	Mean (SD/*IQR*) [95% CI]		
10 ms	0.005 (*0*) [−0.006 to 0.016]	0 (*0*)	0 (*0*)
30 ms	0.039 (*0*) [−0.018 to 0.095]	0 (*0*)	0.025 (*0.02*) [0.009 to 0.042]
50 ms	0.122 (*0.11*) [0.018 to 0.226]	0 (*0*)	0.107 (*0.15*) [0.072 to 0.142]
70 ms	0.306 (*0.24*) [0.151 to 0.461]	0.039 (*0.09*) [0.001 to 0.078]	0.231 (*0.36*) [0.171 to 0.291]
100 ms	0.489 (0.22) [0.367 to 0.611]	0.155 (0.16) [0.056 to 0.254]	0.441 (*0.50*) [0.366 to 0.526]
150 ms	0.750 (0.28) [0.596 to 0.904]	0.458 (0.34) [0.242 to 0.674]	0.683 (0.32) [0.613 to 0.754]
200 ms	0.828 (0.25) [0.688 to 0.968]	0.590 (0.32) [0.373 to 0.807]	0.736 (*0.38*) [0.676 to 0.796]
250 ms	0.873 (0.30) [0.705 to 1.041]	0.637 (*0.57*) [0.405 to 0.869]	0.767 (*0.31*) [0.711 to 0.823]
300 ms	0.770 (0.21) [0.652 to 0.888]	0.721 (0.15) [0.621 to 0.821]	0.743 (0.21) [0.697 to 0.789]
350 ms	0.773 (0.20) [0.664 to 0.882]	0.668 (0.19) [0.540 to 0.796]	0.679 (0.21) [0.633 to 0.726]
400 ms	0.729 (*0.35*) [0.588 to 0.870]	0.603 (0.24) [0.451 to 0.755]	0.653 (0.20) [0.610 to 0.697]
500 ms	0.786 (0.23) [0.660 to 0.912]	0.547 (0.20) [0.419 to 0.675]	0.620 (0.20) [0.576 to 0.665]
700 ms	0.748 (0.20) [0.636 to 0.860]	0.618 (0.26) [0.454 to 0.782]	0.646 (0.21) [0.601 to 0.692]
1,000 ms	0.700 (0.19) [0.597 to 0.803]	0.590 (0.23) [0.445 to 0.735]	0.700 (0.20) [0.657 to 0.743]
The value in italics represents the interquartile range, which is provided in the absence of normally distributed data.			

ISI	Statistical analyses					
	ALS and Co. 1			ALS and Co. 1		
	Statistical test	*p*-value	Effect size	Statistical test	*p*-value	Effect size
10 ms	U-test	*p* = 0.371	*r* = 0.17	U-test	***p* = 0.019**	*r* = 0.24
30 ms	U-test	*p* = 0.197	*r* = 0.25	U-test	*p* = 0.297	*r* = 0.11
50 ms	U-test	***p* = 0.004**	*r* = 0.56	U-test	*p* = 0.869	*r* = 0.02
70 ms	U-test	*p* **= <0.001**	*r* = 0.77	U-test	***p* = 0.046**	*r* = 0.20
100 ms	*t*-test	*p* **= <0.001**	d = 1.72	*t*-test	*p* = 0.608	d = 0.15
150 ms	*t*-test	***p* = 0.021**	d = 0.95	*t*-test	*p* = 0.453	d = 0.21
200 ms	*t*-test	***p* = 0.045**	d = 0.84	U-test	*p* = 0.383	*r* = 0.09
250 ms	*t*-test	*p* = 0.076	d = 0.74	U-test	*p* = 0.168	*r* = 0.14
300 ms	*t*-test	*p* = 0.523	d = 0.26	*t*-test	*p* = 0.650	d = 0.13
350 ms	*t*-test	*p* = 0.183	d = 0.55	*t*-test	*p* = 0.111	d = 0.45
400 ms	*t*-test	*p* = 0.199	d = 0.51	*t*-test	*p* = 0.196	d = 0.37
500 ms	*t*-test	*p* **= 0.009**	d = 1.11	*t*-test	***p* = 0.006**	d = 0.80
700 ms	*t*-test	*p* = 0.153	d = 0.57	U-test	*p* = 0.108	*r* = 0.16
1,000 ms	*t*-test	*p* = 0.178	d = 0.54	*t*-test	*p* = 0.997	d = 0.001

The *p*-values correspond to statistical comparisons between the ALS cohort and age-matched controls (Co 1), and between the ALS cohort and mixed controls (Co 2), as assessed using the *t*-test (*t*) and the Mann–Whitney U test (U). Effect sizes were quantified as Cohen’s d for *t*-tests and r for Mann–Whitney U-tests.

**Table 4:** Overview of the statistics from the two-way ANOVA with repeated measures.

ALS and age-matched controls			ALS and mixedz controls		
Effect	Significance ηp^2^	Interpretation	Effect	Significance ηp^2^	Interpretation
Group	*p* = 0.004 ηp^2^ = 0.31	H-reflex recovery differed significantly between groups	Group	*p* = 0.151 ηp^2^ = 0.02	H-reflex recovery does not differ significantly between groups
ISI	*p* = <0.001 ηp^2^ = 0.73	H-reflex recovery significantly modulated by ISI	ISI	*p* = <0.001 ηp^2^ = 0.62	H-reflex recovery significantly modulated by ISI
Group x ISI	*p* = 0.073 ηp^2^ = 0.08	The groups did not differ in their response patterns across ISIs	Group x ISI	*p* = 0.451 ηp^2^ = 0.01	The groups did not differ in their response patterns across ISIs

The ALS cohort was compared with age-matched controls and mixed controls. The Greenhouse–Geisser correction was applied to account for violations of the sphericity assumption in the repeated measures analysis. Effect sizes are reported as partial eta-squared (ηp^2^).

The analysis of the relationship between H-reflex recovery measurement results and the clinical data of ALS patients using Spearman’s rank correlation revealed no significant correlations. No significant association was observed between the magnitude of recovery at different interval times, the reflex response in the Achilles tendon reflex, the trophic condition of the legs as indicated by visible muscle atrophy, and the cramp index score.

**Figure 2:**
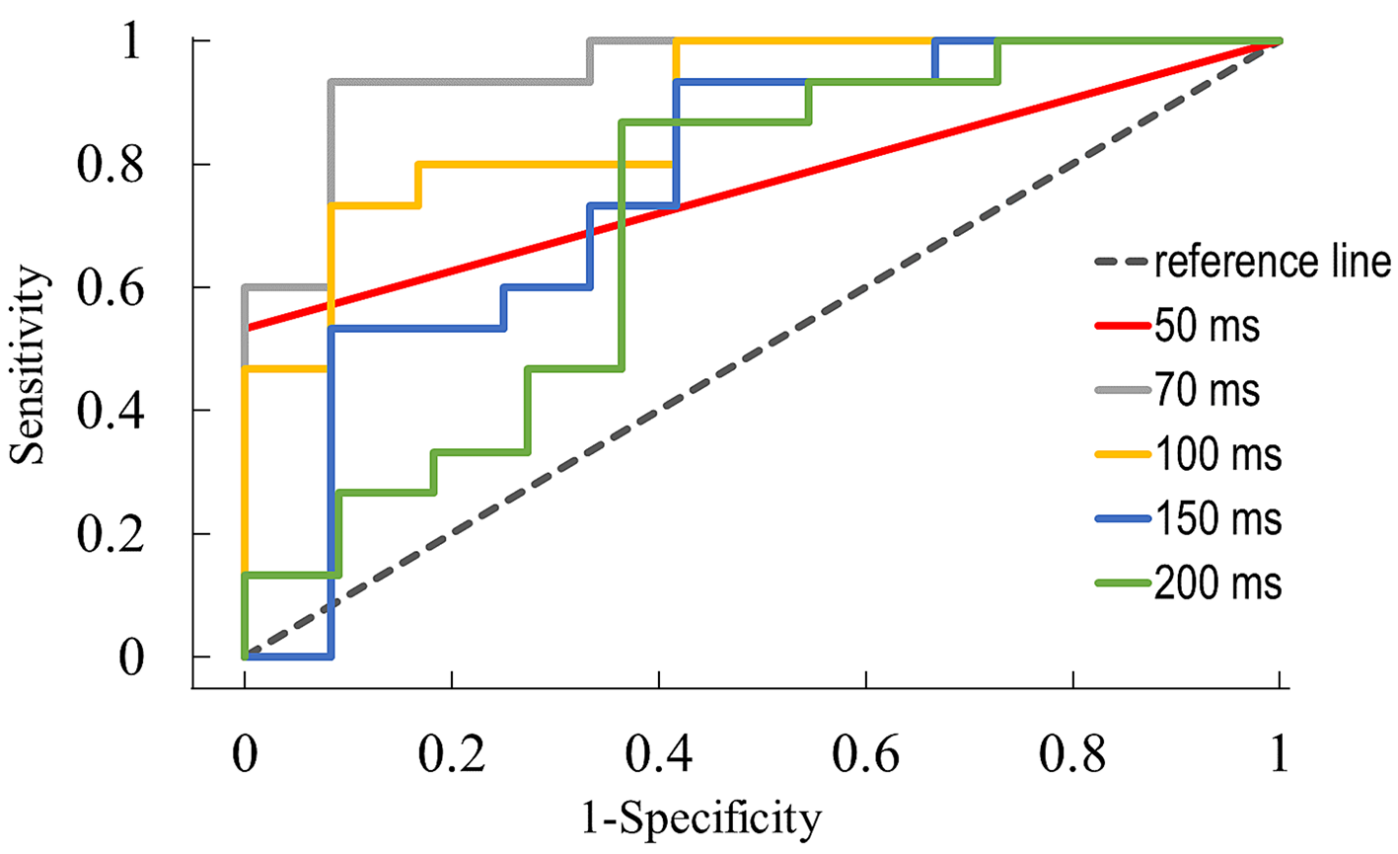
ROC analysis of the H-reflex recovery curve for different interstimulus intervals in ALS patients and individuals from age-matched controls.

A receiver operating characteristic (ROC) analysis was conducted for interstimulus intervals of 50 ms to 200 ms, utilizing data from ALS patients and age-matched controls for comparative evaluation. The highest discriminatory power was observed for the interstimulus interval of 70 ms, with an area under the curve (AUC) of 0.95. With a diagnostic cut-off value of 11.9% recovery, the sensitivity reached 93%, while the 1-specificity was 8%, resulting in a Youden Index of 0.85. The visual representation of the ROC for different interstimulus intervals is shown in Figure 2.

## 4 Discussion

This study provide evidence of altered spinal network activity in patients with ALS demonstrated by the altered H-reflex recovery pattern. The earlier recovery onset and greater magnitude of H-reflex recovery indicate a state of spinal hyperexcitability and a fundamental shift in the balance of inhibitory and excitatory mechanisms within the spinal circuitry in this clinical population.

Our study systematically investigated the recovery curve in ALS patients for ISI up to one second. Significant disparities in the curve pattern compared to age-matched controls were observed, especially during the rapid and dynamic recovery phase. The notably high discriminatory power that was observed in the ROC analysis was a result of group differences in the temporal onset of recovery. At the interstimulus interval of 70 ms with a diagnostic cut-off value of 11.9% recovery, only one of the 15 ALS patients was falsely classified as negative.

The temporal dynamics of H-reflex recovery in ALS have previously been investigated in a limited number of studies, though the findings must be interpreted with caution due to the relatively small sample sizes and specific patient selection criteria. At an interstimulus interval of 1,000 ms, Özyurt et al. found the second H-wave, i.e., that evoked within the period that is considered to be modulated by spinal network activation induced by the first conditioning pulse, to be less reduced in ALS patients with dominant lower extremity involvement compared to healthy controls (ALS: 79.6 ± 30.2% of baseline H-wave amplitude vs. 31.9 ± 18.7% in controls). Only nine ALS – patients with lower extremity involvement and 12 controls were included in this analysis. In contrast, our results gave only non-significant differences between ALS-patients and controls at an interval of 1,000 ms, whereas a clear difference was seen at an ISI of 500 ms in our population (22).

Another work by Raynor et al. compared the inhibitory effects of a prepulse on spinal motoneurons between controls and a preselected population of 12 ALS patients with prominent lower extremity spasticity. Although the applied protocol for assessing spinal network function differed from the one applied here and only one interstimulus interval (10 ms) was tested, the results indicated impaired inhibitory effects of conditioning pulses in ALS patients (23).

In contrast to the cited previous works, our data give insights to the temporal dynamics of altered network inhibition in ALS patients and reveal an earlier recovery onset and increased recovery up to an ISI of 100 ms, suggesting diminished inhibitory function.

A reduction in Renshaw cell activity would explain this finding and might be a direct result of spinal and upper motoneuron degeneration: First, collaterals from several functionally synergistic spinal motoneurons converge to Renshaw cells. Spatial synaptic summation thus is a prerequisite for regular activation of Renshaw cells that is impaired when some of the converging branches are lost due to mototneuron degeneration (41). Second, Renshaw cells are known to be modulated by descending UMN projections that are regularly affected in the course of ALS. Previous studies have shown that voluntary muscle activation reduces inhibition and accelerates recovery, underscoring the significance of central control mechanisms (14). Furthermore, evidence of alterations in descending motor pathways in ALS supports the presence of impaired central regulation (21, 24). These findings highlight the multifaceted nature of neuronal network alterations in ALS, involving both spinal and supraspinal components.

While recurrent inhibition represents a circuit-based modulation, the recovery is also shaped by intrinsic motoneuronal properties such as the afterhyperpolarization (AHP). In ALS, a significant change in AHP duration has been observed. Specifically, in the early stages of the disease, the AHP is shortened (42). Consequently, the relative refractory period following an initial discharge is shortened, allowing the motoneuronal pool to recover its responsiveness more rapidly.

Beyond these postsynaptic and descending influences, the contribution of presynaptic mechanisms must also be considered, particularly due to the use of suprathreshold stimuli in our paradigm. While the early phase of H-reflex recovery is heavily influenced by recurrent inhibition, the later phases are increasingly governed by homosynaptic depression (9, 43). Homosynaptic depression, also known as post-activation depression, is characterized by a reduced neurotransmitter release from previously activated Ia afferents and acts as a fundamental regulatory mechanism for maintaining spinal excitability (44). Since this mechanism is highly rate-sensitive (45), we employed an ISI of 10 s between test trials to minimize the influence of the preceding stimulation. However, in ALS, a significant reduction in the frequency sensitivity of the H-reflex has been documented (22, 26). Such a deficit is typical for conditions involving upper motor neuron dysfunction (46) and would contribute to the earlier and more pronounced recovery observed in our data.

Preclinical studies provide a robust mechanistic foundation for these observations, demonstrating that the Ia-motoneuron junction is compromised well before the onset of overt symptoms (47). Specifically, excitatory synaptic inputs to motor neurons exhibit both reduced efficacy and impaired plasticity, primarily driven by the structural breakdown of the postsynaptic site. Crucially, this synaptic decay is a generalized phenomenon affecting not only proprioceptive Ia-afferents but also descending pathways from the brainstem.

The presence of spinal network dysfunction is supported by recent evidence demonstrating significant alterations in segmental motoneuron excitability and extensive network disinhibition in ALS patients (25). Converging preclinical evidence further indicates that the degeneration of spinal inhibitory interneurons precedes the loss of motor neurons themselves (48). These findings support an emerging consensus that ALS is not solely a disorder of motor neurons but reflects a broader failure of complex spinal network organization.

These findings provide a compelling mechanistic basis for our results. The accelerated and enhanced recovery observed in our cohort likely reflects more than a simple loss of inhibitory control, as it appears to manifest a fundamental loss of synaptic integrity and impaired plasticity. This state is further characterized by diminished homosynaptic depression and potentially altered motoneuronal refractoriness. Such focal alterations at the Ia-motoneuron junction, when combined with broader network-level disinhibition, lead to a state where the spinal cord can no longer maintain stable homeostatic control. Ultimately, this convergence of synaptic and circuit-level deficits results in the pathological hyperexcitability documented in our study.

This dysfunction appears to follow a distinct temporal sequence in which an early loss of inhibitory modulation is followed by the emergence of pathological hyperexcitability as the disease progresses (26). Specifically, the early phase is characterized by a transient increase in heteronymous recurrent inhibition, considered a putative homeostatic response to counteract motoneuron hyperexcitability (49). The subsequent decline into a diminished inhibitory state likely marks the failure of these early compensatory mechanisms, as the progressive loss of motor neurons increasingly compromises network integrity. These observations in humans are consistent with findings from animal models, showing that spinal microcircuits undergo multiphasic homeostatic adaptations aimed at preserving functional output before ultimately decompensating (50). In this context, personalized mapping has revealed participant-specific inhibitory control strategies, reinforcing the marked heterogeneity of ALS (51).

Furthermore, our approach must be distinguished from the dual-pulse framework and multimodal analysis employed by Sangari et al. (52). While both methodologies utilize paired stimulation, they address divergent physiological targets through different experimental lenses. To elucidate the neurophysiological mechanisms underlying H-reflex modifications, Sangari and colleagues integrated H-reflex data with Motor Evoked Potentials (MEPs) to analyze monosynaptic EPSPs of both peripheral and cortical origins. Their findings revealed that in muscles without significant clinical or electrophysiological evidence of peripheral involvement, the H-reflex is notably enhanced while the MEP size is decreased. This suggests a complex dysregulation of motoneuronal recruitment gain and synaptic efficacy (52).

In contrast, our study focuses specifically on the H-reflex recovery curve to characterize the temporal profile of inhibitory recovery, emphasizing the time course of homeostatic failure rather than focusing on recruitment gain. These perspectives are highly complementary, as the interneuronal compensation suggested by Castro et al. (25) and the dysregulation of synaptic dynamics reported by Sangari and colleagues likely provide the micro-circuitry basis for the macro-level breakdown of temporal reflex control observed in our results.

Collectively, these paradigms suggest that spinal network dysfunction in ALS is a multi-dimensional process involving a loss of synaptic precision and interneuronal failure. Our findings thus reflect a fundamental disruption of the spinal cord’s homeostatic balance between excitatory and inhibitory signaling, manifesting as a pervasive failure of temporal inhibitory coordination.

In our study, ALS patients showed a significant increase in M-wave latency, while the H-reflex latency remained stable. A moderate prolongation of motor latencies, up to 125% of the upper limit of normal, is consistent with axonal loss (53). Throughout the course of the disease, large-caliber, fast-conducting alpha motor neurons are selectively lost (54). Since the M-wave relies on these fibers due to their low electrical threshold, their depletion forces the impulse to travel via the remaining, slower-conducting axons, which manifests as increased latency. The stability of the H-reflex latency, despite this peripheral delay, can be attributed to the Henneman size principle (55). The H-reflex primarily recruits smaller motor units that tend to be more resilient and survive longer in ALS than the fastest-conducting fibers. Consequently, the H-reflex continues to utilize these relatively preserved pathways, maintaining a stable latency even as the fastest motor units are lost.

The fundamental dynamics of the H-reflex recovery curve are preserved and comparable in younger healthy individuals, although they manifest at an overall higher level of neural excitability. Reflex levels decrease with increasing age, likely due to multisystemic changes such as reduced motoneuron activity and decreased motonueron excitability (56). Therefore, only a control group of similar age is suitable for comparison with ALS patients.

An elevated reflex level has been identified in the majority of ALS patients through clinical reflex assessments. Despite increased excitability observed in both classical Achilles reflex and H-reflex recovery measurements, no consistent statistical relationship was found between their magnitudes. A possible explanation might be that the ALS-associated involvement of soleus muscle motoneurons that is relevant in H-reflex measurements is only partly correlated with the impairment of gastrocnemius – motoneurons that are also part of the combined response that is observed in clinical Achilles reflex testing.

The presented study has several limitations that should be considered when interpreting our findings: The sample size in the ALS group was relatively small, yet comparable to other studies in this population (22, 23), given the low prevalence of this disorder, ranging from 3.4 to 12.3 per 100,000 individuals (57). In addition, H- and M-wave latencies were not adjusted for body height or segment-specific proportions in this study. Although such normalization can increase precision, it requires specialized equipment, which limits its applicability in everyday clinical practice. Furthermore, all experiments have been performed by a single, unblinded investigator (SW), which have reduced interrater bias, but may have introduced experimenter bias and limits the generalizability of the observations. To compensate for experimenter bias, considerable effort was made in the accurate establishment of the technical procedure, which is documented by the measurement of HRC in a control group that is, to our knowledge, by far larger than those reported in any other study comparing adults with and without ALS. The restriction to only one single time point for measuring individual patients for logistical reasons might be considered another limitation, since reflex findings are known to show considerably intra-individual variability and are thus highly dependent on non-structural changes. Moreover, establishing the reliability of the assessment protocol—comprising both device and protocol-reliability—is warranted in future investigations to substantiate these findings (58). Currently, data on the intraindividual variability in H-reflex-recovery in ALS – patients are lacking. Future studies including both electrophysiological and clinical endpoints are needed to address this issue and clarify the significance of the existing single-time point assessments. Another potential limitation of the current study is the absence of formal adjustments for multiple comparisons. While it is recognized that performing several statistical tests without such corrections increases the potential for Type I errors, there is a substantial methodological discourse regarding this requirement in exploratory research. Some scholars suggest that in studies aimed at hypothesis generation rather than formal confirmation, strict alpha-level adjustments, such as the Bonferroni method, are not strictly required, as they disproportionately increase the risk of Type II errors and may obscure potentially meaningful biological trends (59, 60). Given the exploratory nature of this work and the low prevalence of the disorder, which inherently limits achievable sample sizes and statistical power, we prioritized sensitivity to identify potential patterns. Consequently, these findings provide a valuable foundation for future confirmatory research. Future studies with larger cohorts could benefit from employing linear mixed-effects models. Such an approach would provide enhanced flexibility in modeling individual variability and potential covariates, such as age or height, across the full range of ISIs.

In summary, this study provides further evidence of altered spinal network activity in ALS, characterized by a marked reduction and shortening of reflex inhibition. By utilizing H-reflex recovery testing across multiple ISIs, we can characterize changes within the spinal circuitry and its interaction with modulating inputs that contribute to the disease’s complex pathophysiology. It serves as a valuable non-invasive tool to assess the functional state of the motor system. Further research in larger cohorts is warranted to substantiate its potential as a biomarker for monitoring the progressive network failure involving both upper and lower motor neuron systems characteristic of ALS.

## Data Availability

The raw data supporting the conclusions of this article will be made available by the authors, without undue reservation.
